# The Adaptor Protein SH2B3 (Lnk) Negatively Regulates Neurite Outgrowth of PC12 Cells and Cortical Neurons

**DOI:** 10.1371/journal.pone.0026433

**Published:** 2011-10-18

**Authors:** Tien-Cheng Wang, Hsun Chiu, Yu-Jung Chang, Tai-Yu Hsu, Ing-Ming Chiu, Linyi Chen

**Affiliations:** 1 Institute of Molecular Medicine, National Tsing Hua University, Hsinchu, Taiwan; 2 Department of Life Science, National Tsing Hua University, Hsinchu, Taiwan; 3 Department of Medical Science, National Tsing Hua University, Hsinchu, Taiwan; 4 Institute of Cellular and System Medicine, National Health Research Institutes, Miaoli, Taiwan; Hungarian Academy of Sciences, Hungary

## Abstract

SH2B adaptor protein family members (SH2B1-3) regulate various physiological responses through affecting signaling, gene expression, and cell adhesion. SH2B1 and SH2B2 were reported to enhance nerve growth factor (NGF)-induced neuronal differentiation in PC12 cells, a well-established neuronal model system. In contrast, SH2B3 was reported to inhibit cell proliferation during the development of immune system. No study so far addresses the role of SH2B3 in the nervous system. In this study, we provide evidence suggesting that SH2B3 is expressed in the cortex of embryonic rat brain. Overexpression of SH2B3 not only inhibits NGF-induced differentiation of PC12 cells but also reduces neurite outgrowth of primary cortical neurons. SH2B3 does so by repressing NGF-induced activation of PLCγ, MEK-ERK1/2 and PI3K-AKT pathways and the expression of Egr-1. SH2B3 is capable of binding to phosphorylated NGF receptor, TrkA, as well as SH2B1β. Our data further demonstrate that overexpression of SH2B3 reduces the interaction between SH2B1β and TrkA. Consistent with this finding, overexpressing the SH2 domain of SH2B3 is sufficient to inhibit NGF-induced neurite outgrowth. Together, our data demonstrate that SH2B3, unlike the other two family members, inhibits neuronal differentiation of PC12 cells and primary cortical neurons. Its inhibitory mechanism is likely through the competition of TrkA binding with the positive-acting SH2B1 and SH2B2.

## Introduction

SH2B protein family members, including SH2B1 (SH2-B, PSM), SH2B2 (APS), and SH2B3 (Lnk), are adaptor proteins that regulate several signaling pathways. These family members contain dimerization domain, proline-rich regions, pleckstrin homology (PH), and src homology 2 (SH2) domains. SH2B family members participate in various physiological responses and developmental processes. For metabolic control, SH2B1 and SH2B2 interact with insulin receptor substrate 1 (IRS1), IRS2, or Janus kinase 2 (JAK2) to regulate insulin, leptin, and growth hormone signaling [1], [2], [3], [4], [5], [6]. SH2B1 null mice are obese and develop diabetes [6], [7]. SH2B1 and SH2B2 have also been implicated in neuronal differentiation in PC12 cells and the development of sympathetic neurons [8], [9]. In PC12 cells, nerve growth factor (NGF) binds to the receptor TrkA and activates downstream effectors, such as Shc, phospholipase C gamma (PLCγ, Protein kinase C (PKC), phosphatidylinositol 3-kinases (PI3K)-AKT and the Ras-related mitogen activated protein kinase (MAPK) pathways [10]. The formation of homo- or heterodimers by SH2B1 and SH2B2 through dimerization domain is required for the activation of TrkA [11]. By binding to activated TrkA through its SH2 domain, SH2B1β prolongs TrkA signaling [9]. For cortical neuron development and survival, brain-derived neurotrophic factor (BDNF)-induced PI3K-AKT signaling pathway is required [12], [13], [14]. SH2B1 and SH2B2 are phosphorylated in response to BDNF in cortical neurons [8].

SH2B2 and SH2B3, on the other hand, are known as negative regulators of B cell proliferation [15], [16]. During the development of hematopoietic stem cells, SH2B3 interacts with JAK2 and myeloproliferate leukemia virus oncogene (Mpl) to decrease thrombopoietin-mediated self-renewal [17] through inhibiting signaling pathways including PI3K-AKT, signal transducer and activator of transcription 5 (STAT5), and enhancing p38 MAPK [18]. During stem cell factor (SCF)-mediated mast cell development, SH2B3 serves as a negative regulator which interacts with c-Kit receptor then inhibits downstream ERK1/2 signaling [19]. In tumor necrosis factor-α (TNFα)-mediated inflammatory response of endothelial cells, overexpressing SH2B3 inhibits pERK1/2 and then down-regulates the expression of VCAM-1[20]. Despite the similarity in the domain structure of SH2B3 and other family members, these reports suggest that SH2B3 generally acts as a negative regulator for signaling control. Although a previous study reported that SH2B3 expressed in the brain [21], its role in the brain has not been addressed. In this study, we investigated the role of SH2B3 in neurotrophic factor signaling and neurite outgrowth.

## Materials and Methods

### Animal Handling- Ethics statement

All experiments were conducted in accordance with the guidelines of the Laboratory Animal Center of National Tsing Hua University (NTHU). Animal use protocols were reviewed and approved by the NTHU Institutional Animal Care and Use Committee (Approval number 09837).

### Reagents

2.5 S mouse Nerve growth factor and rat tail collagen I were purchased from BD Bioscience (Franklin Lakes, NJ). Human fibroblast growth factor 1 was purchased from Chingen Inc. (Dublin, OH) and heparin was purchased from Sigma. Protein A sepharose beads was purchased from GE Healthcare bioscience (Piscataway, NJ). Protein G agarose beads, goat anti-SH2B3, rabbit anti-Egr-1, rabbit anti-Tau-1, and rabbit anti-TrkA antibodies were purchased from Santa Cruz Biotechnology (Santa Cruz, CA). TRIzol reagent, Lipofectamine 2000, Alexa Flour 700 goat anti-mouse IgG, and Alexa Fluor 555-conjugated goat anti-mouse IgG secondary antibodies were purchased from Invitrogen (Carlsbad, CA). Profection mammalian transfection system was purchased from Promega (Madison, WI). Rabbit anti-GFP and mouse anti-myc tag antibodies were purchased from Hopegen Biotechnology Development Enterprise (Taipei, Taiwan). Rabbit anti-ERK1/2 antibody and rabbit anti-goat horseradish peroxidase (HRP)-conjugated IgG were purchased from Sigma-Aldrich (St. Louis, MO). Rabbit anti-Akt, mouse anti-STAT3, mouse anti-pERK1/2, mouse anti-phospho-Akt Ser473, rabbit anti-PLCγ, rabbit anti-phospho-PLCγ, and rabbit anti-phospho-STAT3 Ser727 antibodies were purchased from Cell Signaling (Danvers, MA). Rabbit anti-phospho-TrkA Tyr490 antibody was purchased from GeneTex (Irvine, CA).

### Cell lines and cell culture

PC12 and 293T cells were purchased from American Type Culture Collection. PC12 cells were maintained in DMEM containing 10% horse serum (HS), 5% fetal bovine serum (FBS), 1% L-glutamine (L-Gln), 1% antibiotic-antimycotic (AA), and cultured under 10% CO_2_ condition. 293T cells were grown in DMEM containing 10% FBS, 1% L-Gln, 1% AA, and under 5% CO_2_ condition. PC12 cells stably overexpressing GFP (PC12-GFP cells) or GFP-SH2B1β (PC12-SH2B1β cells) were made according to Chen *et al* [22]. PC12 cells stably overexpressing GFP-SH2B3 (PC12-SH2B3 cells) were made by transfecting GFP-SH2B3 to PC12 cells and selecting with medium containing 5 mg/ml of G418 (Invitrogen) for at least 30 days. Pooled populations of stable clones were used to avoid clonal variation.

### Plasmids

pEGFP-C1, pRK5-myc, pEGFP-SH2B1β, myc-SH2B1β and myc-SH2B1β(R555E) were generous gifts from Dr. Christin Carter-Su at University of Michigan, USA [9], [23]. Wild type and R392E mutant of human SH2B3 constructs (in pcDNA3.1) were provided by Dr. Sigal Gery at University of California, Los Angeles School of Medicine, USA [24]. Full length and R392E mutant of SH2B3 were both sub-cloned into pRK5-myc via BamHI/XbaI sites. Full length, R392E mutant, N-terminal region (amino acids 1-296), and C-terminal region of SH2B3 (amino acids 296–575) were also sub-cloned into pEGFP-C1 via BamHI/XbaI, HindIII/XbaI, BamHI/EcoRI, and EcoRI sites respectively. pCMV5-TrkA was purchased from Addgene Inc. (Addgene plasmid 15002).

### Neuronal differentiation and microscopy

PC12 cells were plated on 35-mm culture dishes coated with 0.1 mg/ml of collagen I. For neuronal differentiation, NGF was treated with 50 ng/ml or 100 ng/ml in low-serum medium (DMEM containing 2% FBS, 1% HS, 1% L-Gln, and 1% AA). For FGF1-idncued neuronal differentiation, FGF1 (100 ng/ml) and heparin (10 µg/ml) were mixed in low-serum medium before adding to cells as described in Lin [22]. The definition of morphological differentiation in PC12 cells is that the length of neurites should be at least twice the diameter of the cell body. Images were taken using Zeiss Observer Z1 microscope. Neurite length was measured using Image J software.

### Primary culture of cortical neurons

Brain cortex was dissected from embryonic day 18 (E18) embryos of Sprague-Dawley rats (purchased from BioLASCO Taiwan Co., Ltd.), and treated with papain (10 U/ml) to dissociate cells. Dissociated cells were washed and re-suspended in minimal essential medium (MEM) supplemented with 5% HS and 5% FBS. Neurons were then plated onto dishes coated with 30 µg/ml of poly-L-lysine, and cultured in neurobasal medium with B27 (containing additional 0.025 mM glutamate) on DIV (day *in vitro*) 1. On DIV 3, cells were treated with 5 µM cytosine 1-β-D-arabinofuranoside to inhibit the growth of glial cells. GFP, GFP-SH2B3, GFP-SH2B3(1-296), and GFP-SH2B3(296-575) were transfected to neurons on DIV 4, and then medium was replaced by half of the fresh neurobasal/B27 medium every two days.

### Total RNA purification and semi-quantitative real-time polymerase chain reaction (Q-PCR)

TRIzol reagent was used to isolate total RNA from PC12 cells according to the manufacture's instruction. For reverse transcription, 2 µg of total RNA was converted to cDNA using reverse transcription kit (Applied Biosystems). Q-PCR was performed using SYBR green master mix and ABI Prism 7500 real time PCR system (Applied Biosystems) with specific primers. SH2B3 primer pairs are: forward primer: CTTTCCTTAGTGGCAGAGCC; reverse primer: GACACCCAGAGACCAAGGAT. Glyceraldehyde 3-phosphate dehydrogenase (GAPDH) primer pairs are: forward primer: ATGACTCTACCCACGGCAAGTT; reverse primer: TCCCATTCTCAGCCTTGACTGT. All Q-PCR results were normalized to the levels of GAPDH.

### Knockdown of SH2B3 via RNA interference

pLKO.1 lentiviral vector that contains oligonucleotides (CCGGGACCGGACAGACATCATCTTTCTCGAGAAAGATGATGTCTGTCCGGTCTTTTTG) targeting human SH2B3 sequence (GACCGGACAGACATCATCTTT), pLKO.1-shSH2B3 (Clone number TRCN0000116286), was obtained from National Core Facility at the Institute of Molecular Biology, Genomic Research Center, Academic Sinica, Taiwan. Lentivirus containing pLKO.1-shSH2B3 was prepared from 293T cells co-transfected with pLKO.1-shSH2B3, pCMVΔR8.91, and pMD.G. Medium containing lentivirus was harvested 24 and 48 h after transfection and added into PC12 cells in the presence of polybrene (10 µg/ml). PC12 cells were then subjected to puromycin (5 µg/ml) selection for at least 2 days.

### Immunofluorescence and immunohistochemistry

For PC12 cells immunofluorescence, cells were plated on 35-mm culture dishes coated with collagen I. After indicated treatment, cells were fixed by 4% of paraformaldehyde (PFA), and permeabilized by 0.1% of Triton X-100. Cells were then incubated in blocking solution containing 1% bovine serum albumin, followed by the incubation of indicated antibodies and mounted with Prolong Gold reagent (Invitrogen). Images were taken using Zeiss Observer Z1 microscope. E18 rat embryo brains were fixed by 4% of PFA and then incubated in 30% sucrose overnight for dehydration. Before embedding, rat brains were incubated in 50% O.C.T. (optimal cutting temperature) compound mixed with 30% sucrose/PBS at room temperature for 2 hours. Rat brains were immersed in 100% O.C.T. compound and embedded with liquid nitrogen. The embedded tissues were conserved in −80°C before section. Embedded tissues were sectioned by cryostat microtome (MICROM HM550) under −20 to −30°C, each section was 10 µm thick and picked up on a glass slide. The slides were air-dried by placing at room temperature in ventilator and then preserved in −20°C. Immunocytochemistry images of rat brain slices were taken using Zeiss LSM 510 confocal microscope.

### Immunoblotting and immunoprecipitation

Cells were treated as indicated and lysates were collected in RIPA buffer (50 mM Tris-HCl, pH 7.5, 150 mM NaCl, 2 mM EGTA, 1% Triton X.100) containing protease inhibitors (1 mM Na_3_VO_4_, 10 ng/ml leupeptin, 10 ng/ml aprotinin, 1 mM phenylmethylsulfonyl fluoride, PMSF). Proteins were resolved by sodium dodecyl sulfate-polyacrylamide gel electrophoresis (SDS-PAGE) and then immobilized to nitrocellulose membrane for western blotting analysis using the indicated antibodies. The immunoblots were detected using either IRDye-conjugated IgG and the Odyssey Infrared Imaging System (LI-COR Biosciences, Lincoln, NE) or HRP-conjugated IgG and ECL system. For immunoprecipitation, cell lysates were incubated with the indicated antibodies and then Protein A sepharose or Protein G agarose beads. Beads were washed with pre-chilled lysis buffer and then boiled in Laemmli sample buffer. The immunoprecipitated proteins were analyzed by western blotting.

### Statistics

Q-PCR, neuronal differentiation, and immunoblotting quantification results were expressed as mean ± standard error and performed Paired Student's t-test. Significance (*) was defined as *P*<0.05. Quantification results of neurite length were expressed as mean ± standard deviation and performed one-way ANOVA. Significance (*) was defined as *P*<0.05.

## Results

### SH2B3 is expressed in rat brain cortex and is a NGF-induced gene

**Figure 1:**
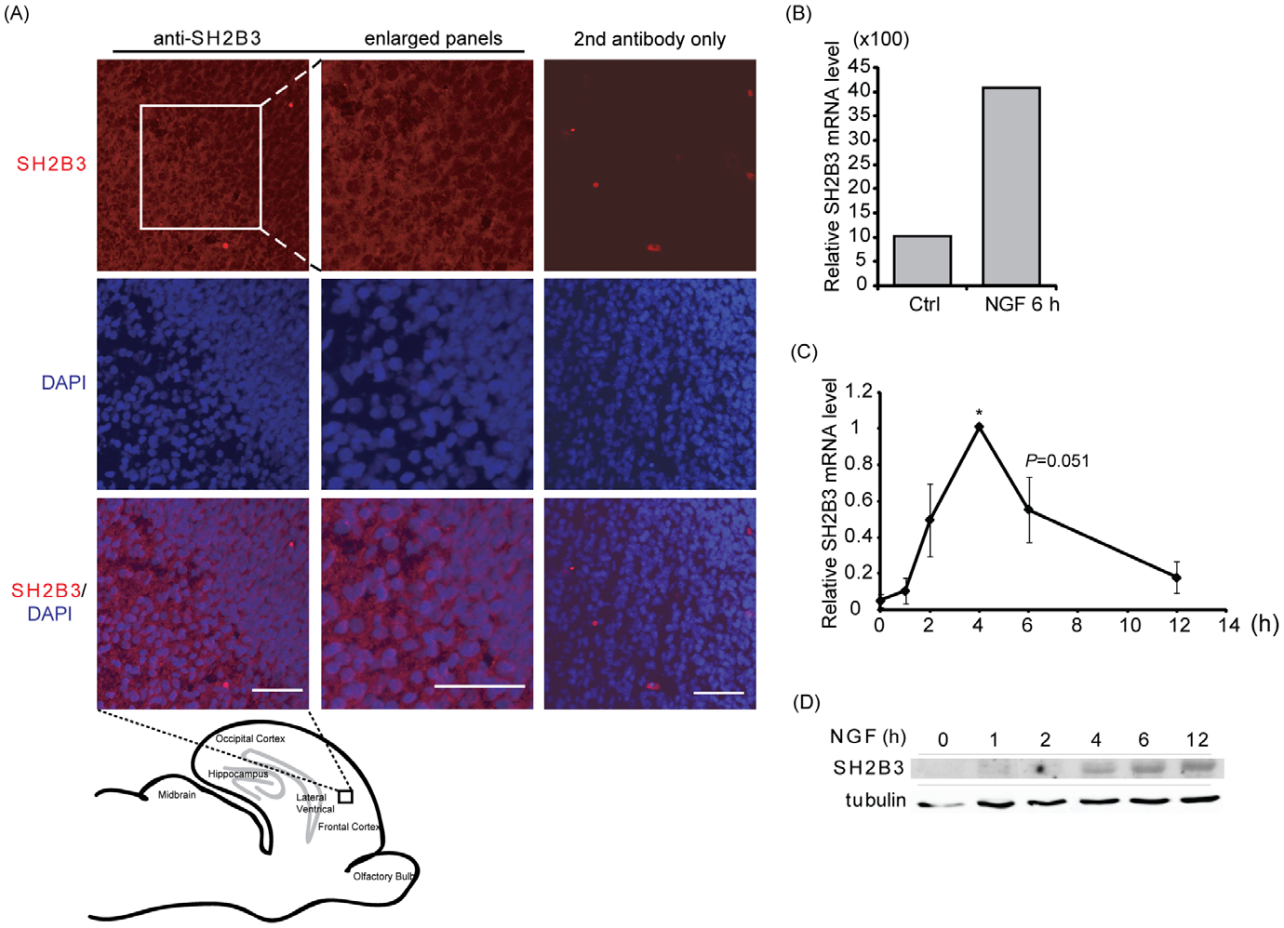
Expression of SH2B3 in the E18 rat brain cortex and in PC12 cells. (A) Brain slices of E18 rats were subjected to immunofluorescence staining using anti-SH2B3 antibody followed by Alexa Fluor 555-conjugated secondary antibody (shown in red). Enlarged panels from the square box area are shown in the middle column. Secondary antibody controls (without primary antibody) are shown in the panels on the right column. DAPI staining in blue shows the localization of the nucleus. Images were taken using Zeiss LSM 510. Scale bar: 50 µm. (B) Microarray results generated from rat affymetrix chips. Relative mRNA expressions of SH2B3 in PC12-GFP cells treated without (Ctrl) or with 100 ng/ml NGF for 6 h are shown. (C) Relative mRNA expressions of SH2B3 in PC12 cells treated with NGF 50 ng/ml for the indicated time periods were analyzed using Q-PCR. The relative SH2B3 levels were normalized to the levels of GADPH. *: P<0.05, paired Student t-test. (D) Protein lysates from PC12 cells treated with NGF 50 ng/ml for indicated time period were immunoblotted using anti-SH2B3 and anti-tubulin antibodies. Tubulin levels were used as loading controls.

To investigate the neuronal role of SH2B3, we set out to determine whether SH2B3 is expressed in the central nervous system. The presence of SH2B3 in brain slices from E18 rat embryos was determined via immunofluorescence staining. As revealed in Figure 1A, SH2B3 was robustly expressed in the cerebral cortex of brain. To understand whether SH2B3 participates in the development of nervous system, we used PC12 cells, a well-documented neuronal model system, to address the roles of SH2B3 in neuronal differentiation. PC12 cells can be differentiated into sympathetic-like neurons in the presence of neurotrophic factors such as NGF and FGF1 [25], [26]. Based on our previous microarray data, the relative mRNA levels of SH2B3 was increased four-fold in response to 6 h of NGF stimulation (Figure 1B). Q-PCR analysis was performed to confirm the microarray data. The relative mRNA levels of SH2B3 were increased as early as 2 h after NGF addition, peaked at 4 h, remained high at 6 h and reduced to near basal level by 12 h (Figure 1C). The NGF-induced SH2B3 protein levels were obviously increased by 4 h of NGF treatment and continued to increase to at least 12 h. Although mRNA levels of SH2B3 were reduced by 12 h, the protein levels remained high. These results suggest that NGF-induced SH2B3 proteins are quite stable and may function not only during initiation phase but also during neurite elongation.

### Overexpressing SH2B3 inhibits and reducing SH2B3 increases NGF-induced differentiation of PC12 cells

**Figure 2:**
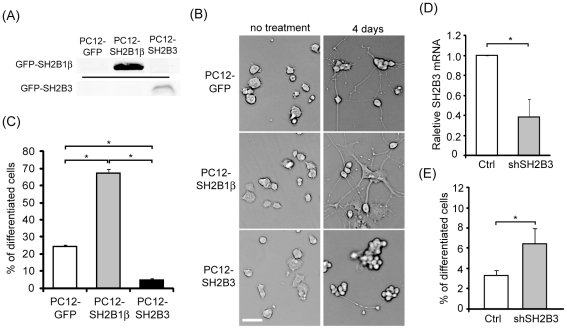
NGF-induced neuronal differentiation of PC12 cells stably expressing GFP, GFP-SH2B1β or GFP-SH2B3, and SH2B3-knockdown cells. (A) Cell lysates were extracted from PC12-GFP, PC12-SH2B1β or PC12-SH2B3 cells and analyzed via western blotting using anti-GFP antibody. (B) PC12-GFP, PC12-SH2B1β or PC12-SH2B3 cells were treated without or with NGF 50 ng/ml for 4 days. Representative images of live cells are shown. Scale bar: 50 µm. (C) Differentiation percentage of PC12-GFP, PC12-SH2B1β or PC12-SH2B3 cells treated with NGF 50 ng/ml for 4 days. The definition of differentiation is shown in the Materials and Methods. *: P<0.05, paired Student t-test. (D) PC12 cells were infected with (shSH2B3) or without (Ctrl) lentivirus containing shSH2B3, followed by 50 ng/ml NGF treatment for 4 h. Relative mRNA of SH2B3 was analyzed using Q-PCR. The relative SH2B3 levels were normalized to the levels of GADPH. *: P<0.05, paired Student t-test. (E) PC12 cells were infected with shSH2B3-containing lentivirus (shSH2B3) or without (Ctrl), and then treated with 100 ng/ml NGF for 24 h. Percentages of differentiation were determined and shown. *: P<0.05, paired Student t-test.

In order to perform biochemical studies to determine the role of SH2B3 during neuronal differentiation, three stable cell lines – PC12 cells overexpressing GFP (PC12-GFP cells), GFP-SH2B1β (PC12-SH2B1β cells) or GFP-SH2B3 (PC12-SH2B3 cells) were established. The expressions of SH2B1β and SH2B3 were confirmed using western blotting (Figure 2A). To determine their effect on neuronal differentiation, PC12-GFP, PC12-SH2B1β and PC12-SH2B3 cells were treated with or without NGF for 4 days. Consistent with previous results, PC12-SH2B1β cells enhanced neurite outgrowth compared to PC12-GFP cells. In contrast, PC12-SH2B3 cells dramatically reduced neurite outgrowth (Figure 2B). The percentage of morphological differentiation was determined as described in the Materials and Methods. As shown in Figure 2C, approximately 25% of PC12-GFP cells were differentiated, whereas more than 65% of PC12-SH2B1β cells and less than 5% of PC12-SH2B3 cells were differentiated in response to 50 ng/ml NGF for 4 days. Level of SH2B3 was reduced via RNA interference approach. Lentivirus that contain shSH2B3 were used to infect PC12 cells. Level of SH2B3 mRNA was determined via Q-PCR. As shown in Figure 2D, shSH2B3 reduced mRNA of SH2B3 to 40%. Knocking down SH2B3 enhanced NGF-induced neuronal differentiation (Figure 2E). These results demonstrated that overexpressing SH2B3 inhibited, whereas reducing SH2B3 increased, NGF-induced neuronal differentiation.

### SH2B3 down-regulates NGF- and FGF1-induced signaling

**Figure 3:**
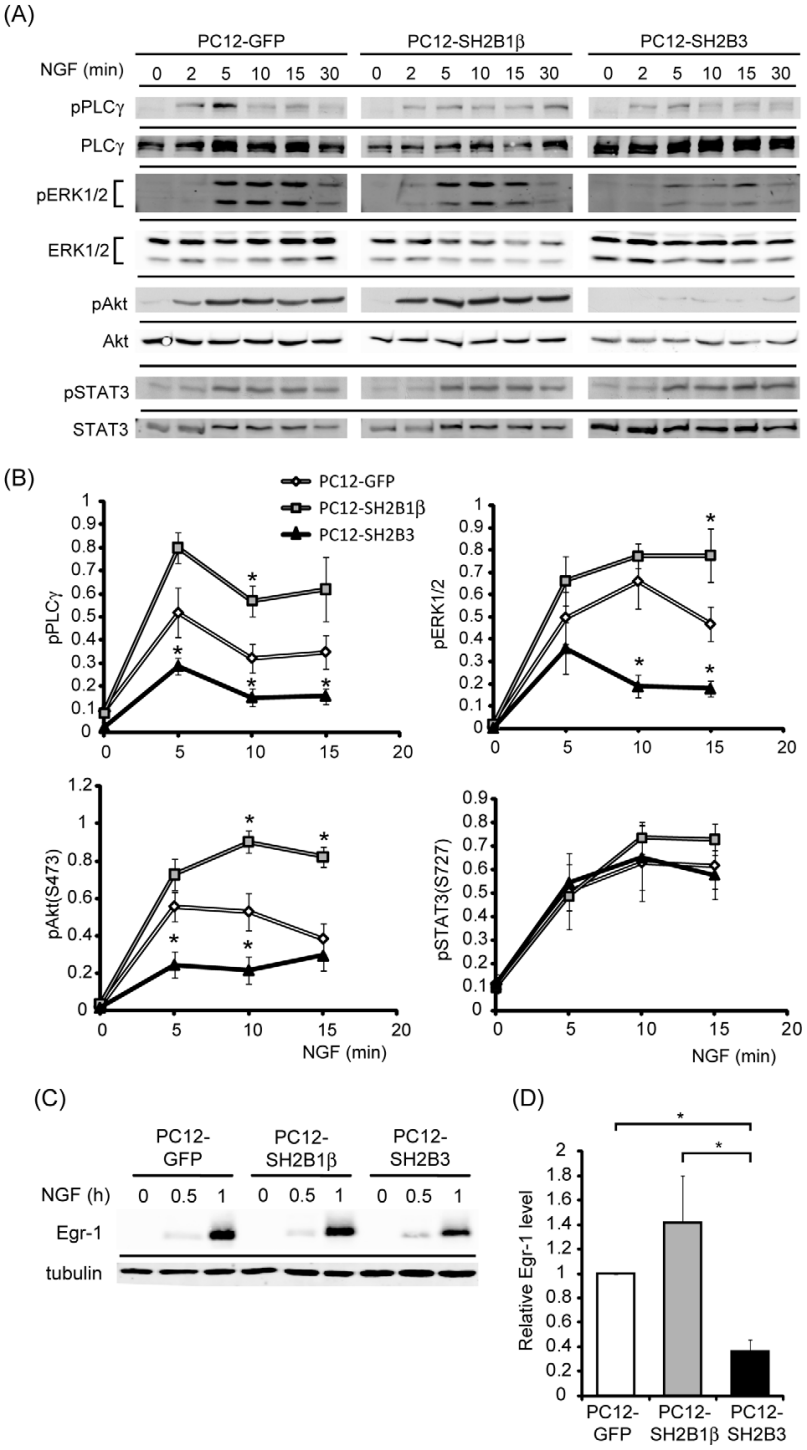
NGF-induced signaling in PC12-GFP, PC12-SH2B1β and PC12-SH2B3 cells. (A) PC12-GFP, PC12-SH2B1β and PC12-SH2B3 cells were cultured in serum-free medium overnight before treating with 100 ng/ml NGF for the indicated time points. Cell lysates were collected and analyzed via western blotting using anti-pPLCγ, anti-PLCγ, anti-pERK1/2, anti-ERK1/2, anti-phospho-Ser473 of AKT (pAkt), anti-AKT, anti-phospho-Ser727 of STAT3 (pSTAT3) and anti-STAT3 antibodies. (B) The relative levels of pPLCγ, pERK1/2, pAKT, or pSTAT3 were normalized to levels of PLCγ, ERK1/2, AKT and STAT3 from three independent experiments. *: P<0.05, paired Student t-test. (C) PC12-GFP, PC12-SH2B1β and PC12-SH2B3 cells were treated with 100 ng/ml of NGF for 0, 0.5 or 1 h. Cell lysates were subjected to western blot analysis using anti-Egr-1 or anti-tubulin antibody. Tubulin levels serve as loading controls. (D) The relative levels of 1 h NGF-induced Egr-1 from three independent experiments were normalized to those for PC12-GFP cells ( = 1). *: P<0.05, paired Student t-test.

Given that SH2B family members are signaling adaptors, the effect of overexpressing SH2B3 on neurotrophic factor signaling was investigated. NGF is known to activate pathways involving PLCγ, MEK-ERK1/2 and PI3K-AKT. Thus, neurotrophic factors-induced phosphorylations of PLCγ, ERK1/2 and AKT were compared among PC12-GFP, PC12-SH2B1β and PC12-SH2B3 cell lines. In response to NGF, pPLCγ, pERK1/2 and pAKT were induced within 2-5 min. The relative levels of pPLCγ were increased in PC12-SH2B1β cells and reduced in PC12-SH2B3 cells compared to PC12-GFP cells. Phosphorylation of ERK1/2 was not obviously different between PC12-GFP and PC12-SH2B1β cells whereas pERK1/2 levels were reduced in PC12-SH2B3 cells compared to PC12-GFP cells. Phosphorylation of AKT was higher in PC12-SH2B1β and lower in PC12-SH2B3 cells compared to PC12-GFP cells (Figure 3A-B). Previous studies suggest that STAT3 functions downstream of ERK1/2 during NGF and FGF1 signaling [22], [27]. However, NGF-induced pSTAT3 levels were not obviously different among these three stable cell lines (Figure 3A–B). The expression of a NGF-responsive gene, Egr-1, was also determined. As shown in Figure 3C, the expression of Egr-1 was induced within 1 h after the addition of NGF. While overexpression of SH2B1β did not affect the expression of Egr-1, overexpression of SH2B3 reduced it (Figure 3C–D). These results demonstrate that overexpression of SH2B3 represses NGF signaling including the activation of PLCγ, ERK1/2, AKT and the expression of Egr-1.

**Figure 4:**
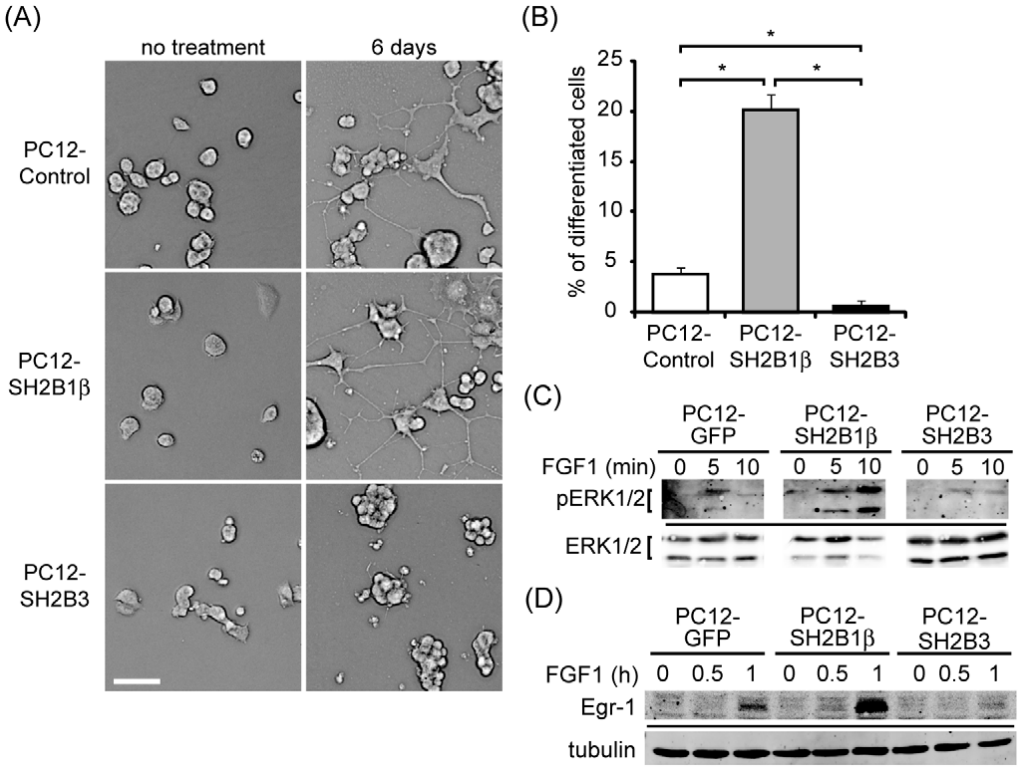
Overexpressing SH2B3 inhibits FGF1-induced signaling and neuronal differentiation of PC12 cells. (A) PC12, PC12-SH2B1β and PC12-SH2B3 cells were treated with 100 ng/ml of FGF1 together with 10 µg/ml of heparin for 6 days. Representative images of live cells are shown. Scale bar: 50 µm. (B) PC12 cells were treated as in (A), differentiation rate of PC12, PC12-SH2B1β, or PC12-SH2B3 cells on Day 6 was analyzed as described in the Materials and Methods from three independent experiments. *: P<0.05, paired Student t-test. (C) PC12-GFP, PC12-SH2B1β and PC12-SH2B3 cells were treated with 100 ng/ml of FGF1 together with 10 µg/ml of heparin for 0, 5, or 10 min. Cell lysates were collected, analyzed via western blotting using anti-pERK1/2 and anti-ERK1/2 antibodies. (D) PC12-GFP, PC12-SH2B1β and PC12-SH2B3 cells were treated with 100 ng/ml of FGF1 together with 10 µg/ml of heparin for 0, 0.5, or 1 h. Cell lysates were analyzed via western blotting using anti-Egr-1 and anti-tubulin antibodies. Tubulin levels serve as loading controls.

To determine whether SH2B3 inhibits neuronal differentiation in neurotrophic factor-specific manner, its effects on FGF1-induced neurite outgrowth and signaling were also addressed. Developmental response to FGF1 in PC12 cells is slower than that to NGF, thus PC12, PC12-SH2B1β, and PC12-SH2B3 cells were subjected to FGF1 stimulation for 6 days. PC12-SH2B1β cells clearly enhanced FGF1-induced neurite outgrowth compared to PC12 and PC12-SH2B3 cells inhibited it (Figure 4A). Neurite outgrowth of PC12-SH2B1β cells was obvious within 2 days of FGF1 treatment whereas no neurite initiation was found for PC12-SH2B3 cells even after 7 days (data not shown). The percentages of differentiation for PC12 control, PC12-SH2B1β, and PC12-SH2B3 cells were in average 4, 20, and 1% respectively (Figure 4B). FGF1-induced pERK1/2 and expression of Egr-1 were enhanced in PC12-SH2B1β cells and reduced in PC12-SH2B3 cells (Figure 4C–D). Together with the data from Figure 3, these results suggest that overexpression of SH2B3 inhibits both NGF- and FGF1-induced signaling and neurite outgrowth.

### SH2B3 reduces the interaction between SH2B1β and TrkA

**Figure 5:**
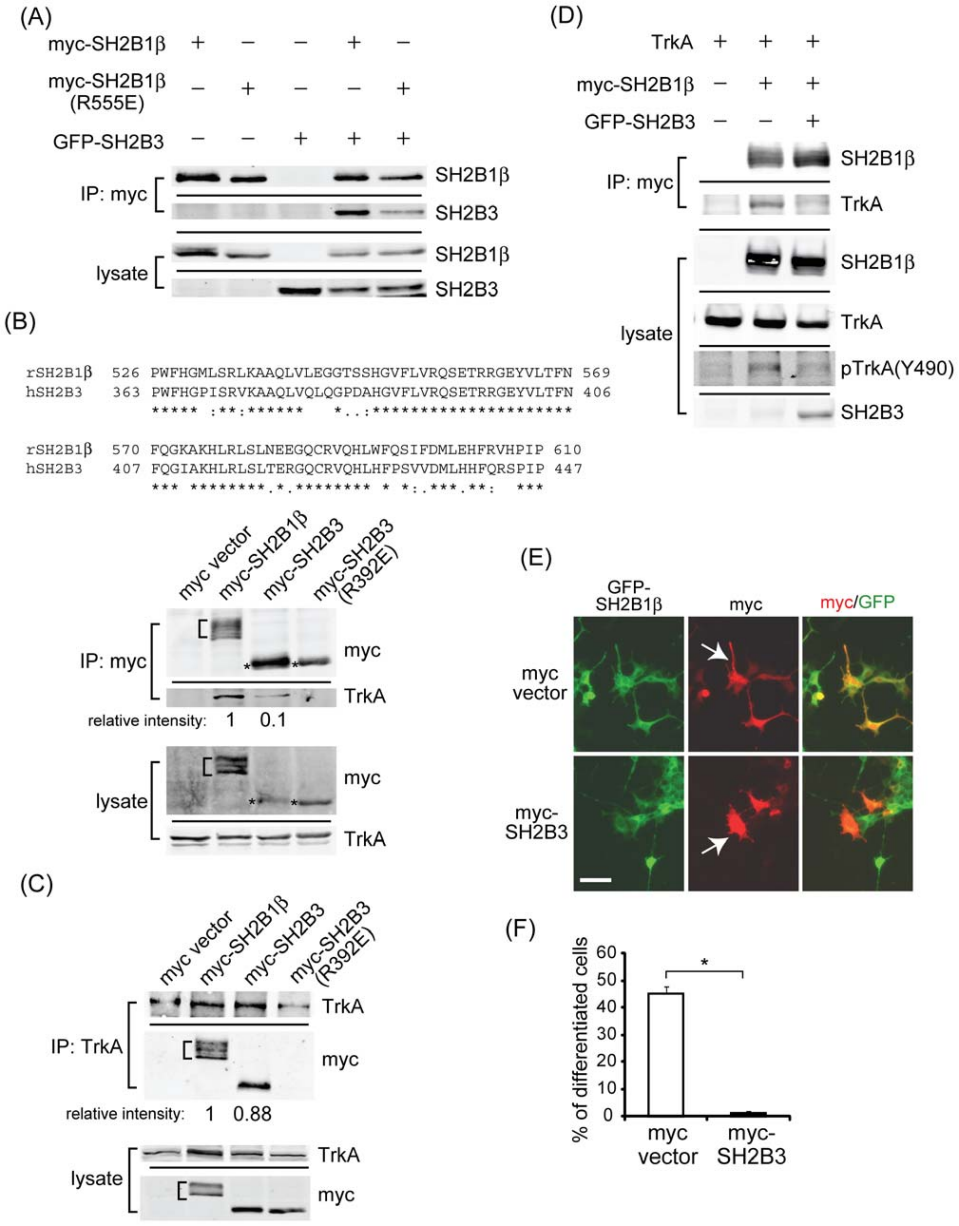
Overexpression of SH2B3 interferes with the interaction between SH2B1β and TrkA. (A) PC12 cells were transfected with myc-SH2B1β, myc-SH2B1β(R555E), or GFP-SH2B3. Cell lysate was immunoprecipitated using anti-myc antibody and immunoblotted with anti-myc and anti-GFP antibodies. Lower panels are cell lysate controls. (B) 293T cells were transfected with pCMV5-TrkA, and co-expressed with myc vector, myc-SH2B1β, myc-SH2B3, or myc-SH2B3(R392E) followed by 100 ng/ml NGF treatment for 15 min. Cell lysates were immunoprecipitated using anti-myc antibody and immunoblotted with anti-myc and anti-TrkA antibodies. Lower panels are cell lysate controls. (C) 293T cells were treated as in (B), cell lysates were immunoprecipitated using anti-TrkA antibody and immunoblotted with anti-TrkA and anti-myc antibodies. Lower panels are cell lysate controls. (D) 293T cells were transfected with pCMV5-TrkA, myc-SH2B1β and/or GFP-SH2B3 followed by 100 ng/ml NGF treatment for 15 min. Cell lysates were immunoprecipitated using anti-myc antibody and immunoblotted with anti-TrkA and anti-myc antibodies. Lysate blots were immunoblotted with anti-myc, anti-TrkA, anti-phospho-tyrosine 490 of TrkA [pTrkA(Y490)], and anti-GFP antibodies (lower panels). (E) PC12-SH2B1β cells were transiently transfected with myc vector or myc-SH2B3. Cells were then treated with 100 ng/ml of NGF for 24 h and then subjected to immunofluorescence staining using anti-myc antibody and Alexa Fluor 555-conjugated secondary antibody (red). Green fluorescence represents GFP-SH2B1β. Images were taken using Zeiss Observer Z1. Scale bar: 50 µm. (F) PC12-SH2B1β cells were treated as in (E), percentages of differentiation for myc-positive cells on were analyzed as described in the Materials and Methods from three independent experiments. *: P<0.05, paired Student t-test.

We next determine whether SH2B3 affects the functions of SH2B1β and TrkA. SH2B1β and SH2B2 can form homo- or heteromultimers through the conserved dimerization domain (DD) [28], and bind to TrkA through SH2 domain [9]. SH2B3 also contains the conserved DD and SH2 domains. Thus, it is possible that SH2B3 may form heteromultimers with SH2B1 and may interact with TrkA via SH2 domain. To this end, GFP-SH2B3 was co-expressed with either myc-SH2B1β or myc-SH2B1β(R555E) of which SH2 domain was mutated and cannot interact with TrkA. Protein-protein interaction was determined by co-immunoprecipitation experiments. As shown in Figure 5A, GFP-SH2B3 was co-immunoprecipitated with both SH2B1β and SH2B1β(R555E). This result suggests that SH2B3 is capable of interacting with SH2B1β, likely through DD domain, whereas SH2 domain of SH2B1β is not required for the interaction with SH2B3. Based on the similarity of the SH2 domain between SH2B1β and SH2B3 (Figure 5B), we anticipate that SH2B3 would bind to TrkA. To this end, vector control, myc-SH2B1β, myc-SH2B3, or myc-SH2B3(R392E), together with TrkA construct, were co-transfected to 293T cells followed by NGF stimulation for 15 min. SH2B1β and SH2B3 were immunoprecipitated via anti-myc antibody, the relative amount of TrkA in SH2B3-containing complexes was 10% of that in SH2B1β-containing complexes (Figure 5B). This result suggests that most of the overexpressed SH2B3 may form homodimers by itself, heterodimers with SH2B1, or in other protein complexes. No interaction between SH2B3(R392E) and TrkA was detected. Interestingly, when immunoprecipitated TrkA, the relative amount of SH2B3 in TrkA-containing complexes was 88% of SH2B1β (Figure 5C). Similarly, no interaction between TrkA and SH2B3(R392E) was found. These results suggest that, although relatively low level of overexpressed SH2B3 binds to TrkA, the relative amounts of SH2B3 and SH2B1β in TrkA-containing complexes are comparable.

Once NGF binds to TrkA receptor, TrkA dimerizes and its cytoplasmic region is tyrosine phosphorylated. SH2B1β has been shown to bind to activated TrkA and positively enhances tyrosine phosphorylation of TrkA to prolong downstream signaling pathway [8], [9]. The fact that SH2B3 could bind to both SH2B1β and TrkA suggests that SH2B3 would affect SH2B1β-mediated NGF signaling and neurite outgrowth. To test this hypothesis, combinations of TrkA, myc-SH2B1β and GFP-SH2B3 were transfected to 293T cells followed by NGF stimulation. As revealed in the lysate, phosphorylation of TrkA at tyrosine 490 [pTrkA(Y490)] was increased when TrkA and SH2B1β were overexpressed. pTrkA(Y490) recruits another adaptor protein Shc and the levels of TrkA(Y490) is positively correlates with SH2B1-mediated NGF signaling [8]. In contrast, as SH2B3 was also co-expressed together with TrkA and SH2B1β, the level of pTrkA(Y490) was reduced (Figure 5D, lower panels). This results suggest that overexpression of SH2B3 may affect TrkA activation, possibly through sequestering away SH2B1β from TrkA or competing for TrkA binding with SH2B1β. As predicted, the interaction between TrkA and SH2B1β was significantly reduced when SH2B3 was also co-expressed (Figure 5D, upper panels). To further examine whether SH2B3 would functionally reduce SH2B1β-enhanced neuronal differentiation, PC12-SH2B1β cells were transiently transfected with SH2B3 and NGF-induced neurite outgrowth was determined. As shown in Figure 5E, neurite outgrowth in PC12-SH2B1β cells that transiently overexpressed myc-SH2B3 (white arrows) was inhibited compared to non-transfected or vector-transfected cells. Quantified results showed that overexpressing SH2B3 in PC12-SH2B1β cells significantly inhibited neuronal differentiation (Figure 5F). Together, these results suggest that SH2B3 could inhibit NGF-induced signaling and neurite outgrowth through (1) interacting with SH2B1β and sequestering it away from TrkA binding; and/or (2) competing with SH2B1β for TrkA binding.

### Carboxyl-termini but not amino-termini of SH2B3 is required for inhibiting NGF-induced neurite initiation of PC12 cells

**Figure 6:**
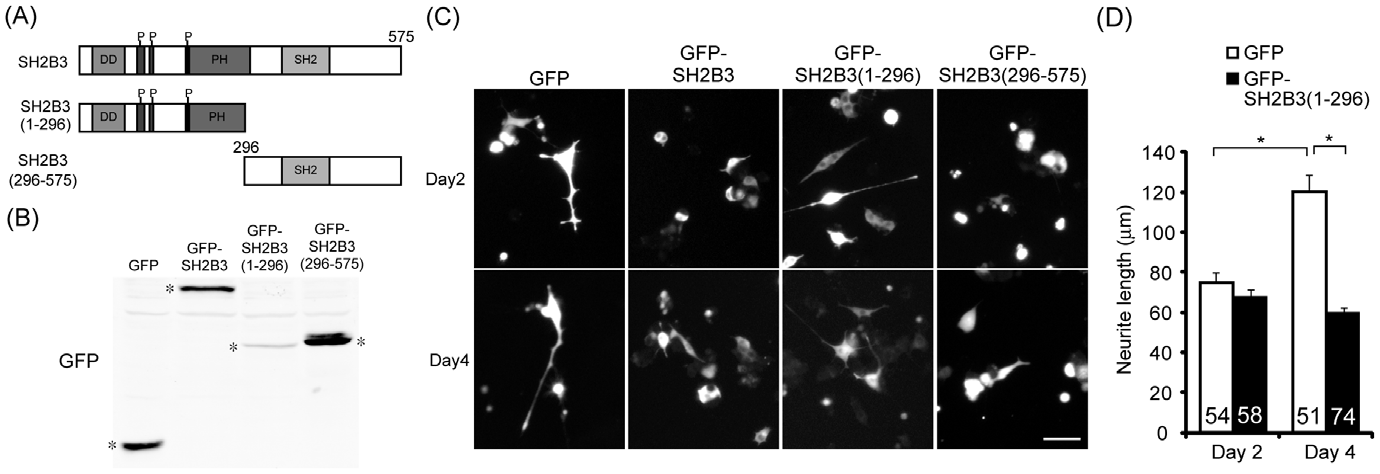
Carboxyl-terminal region of SH2B3 is sufficient to inhibit NGF-induced neurite initiation of PC12 cells. (A) Schematic representation of full-length and truncated human SH2B3 proteins. P represents proline-rich domain. (B) PC12 cells were transiently transfected with GFP, GFP-SH2B3, GFP-SH2B3(1–296), or GFP-SH2B3(296–575). Cells lysates were analyzed via western blotting using anti-GFP antibody. * indicates the overexpressed GFP-fusion proteins. (C) PC12 cells were transiently transfected with GFP, GFP-SH2B3, GFP-SH2B3(1–296), or GFP-SH2B3(296–575) followed by the treatment of 100 ng/ml of NGF for 2 or 4 days. Representative images of live cells were shown. Scale bar: 50 µm. (D) PC12 cells were treated as in (C), the averaged neurite length on Day 2 and 4 was measured from three independent experiments. Total numbers of cells counted are shown on the bars. *: P<0.05, one-way ANOVA.

**Figure 7:**
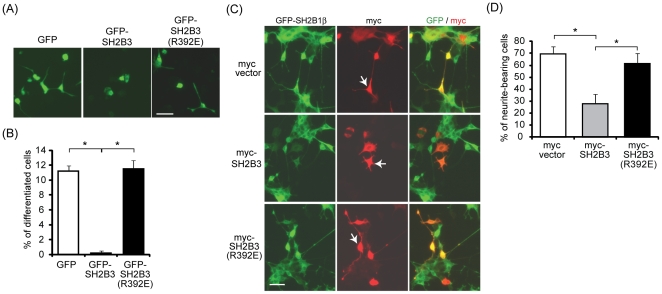
SH2 domain of SH2B3 is required for inhibiting neuronal differentiation in PC12 cells. PC12 cells were transiently transfected with GFP, GFP-SH2B3, or GFP-SH2B3(R392E) and then treated with 100 ng/ml of NGF for 2 days. Representative images of live cells are shown. Scale bar: 50 µm. (B) PC12 cells were treated as in (A), differentiation rate of GFP-, GFP-SH2B3, or GFP-SH2B3(R392E)-expressing cells on Day 2 was analyzed as described in the Materials and Methods from three independent experiments. *: P<0.05. (C) PC12-SH2B1β cells were transiently transfected with myc vector (upper panels), myc-SH2B3 (middle panels), or myc-SH2B3(R392E) (lower panels). Cells were then treated with 100 ng/ml of NGF for 24 h followed by immunofluorescence staining using anti-myc antibody and Alexa Fluor 555-conjugated secondary antibody (red). Green fluorescence represents GFP-SH2B1β. Images were taken using Zeiss Observer Z1. Scale bar: 50 µm. (D) PC12-SH2B1β cells were treated as in (C), percentages of myc-positive cells that have neurites were determined from three independent experiments. *: P<0.05, paired Student t-test.

To distinguish which mechanism accounts for the inhibitory effect of SH2B3 on neurite outgrowth, amino (N)- and carboxyl (C)-terminal truncation mutants were constructed. SH2B3(1–296) contains DD, proline-rich and PH domains. SH2B3(296–575) contains SH2 domain (Figure 6A). If SH2B3 acts to bind and sequester SH2B1β away from binding to TrkA, expressing SH2B3(1–296) should be sufficient to block NGF-induced neurite outgrowth. If SH2B3 and SH2B1β compete for TrkA binding, expressing SH2B3(296–575) shall inhibit neurite outgrowth. The expression of these truncation mutants is shown in Figure 6B. To determine their effect on neurite outgrowth, PC12 cells were transiently transfected with GFP, GFP-SH2B3, GFP-SH2B3(1-296), and GFP-SH2B3(296–575) followed by NGF treatment. In control PC12-GFP cells, neurites were initiated within 2 days and the length of neurites increased on Day 4. In line with the results from Figures 2, 4 and 5, overexpression of SH2B3 inhibited neurite outgrowth. Neurite initiation (Day 2) was not affected by overexpression of SH2B3(1–296) but neurite elongation (Day 4) appeared to be impaired. Overexpression of SH2B3(296–575), on the other hand, inhibited neurite initiation (Figure 6C). The averaged length of neurites for cells transfected with GFP or GFP-SH2B3(1–296) was 75 µm on Day 2 of NGF treatment whereas the length of neurites increased to 120 µm for GFP-transfected cells, and the length of neurites for GFP-SH2B3(1–296)-transfected cells did not increase (Figure 6D). These results suggest that the SH2 domain of SH2B3 is responsible for the main inhibitory effect of SH2B3 on neurite initiation. In other words, SH2B3 likely competes with SH2B1β for TrkA binding. If this is true, one would expect the dominant negative mutant of SH2B3 that does not bind to receptor tyrosine kinase would not block neurite outgrowth. R392E mutation in the SH2 domain of SH2B3 prevents its binding to c-Kit [19]. To this end, PC12 cells were transiently transfected with GFP, GFP-SH2B3, or GFP-SH2B3(R392E), as shown in Figure 7A. Cells expressing GFP or GFP-SH2B3(R392E) exhibited neurites after 2 days of NGF treatment. In contrast, cells expressing SH2B3 inhibited neurite initiation (Figure 7A). Similarly, around 11% of cells expressing GFP or GFP-SH2B3(R392E) were differentiated whereas cells expressing SH2B3 inhibited neuronal differentiation (Figure 7B). Overexpression of SH2B3 in PC12-SH2B1β cells interfered with SH2B1β-enhanced neurite outgrowth whereas overexpression of SH2B3(R392E) did not (Figure 7C). Percentage of neurite-bearing cells for PC12-SH2B1β cells was 70%. When SH2B3 was co-expressed, the percentage was reduced to around 30% whereas overexpression SH2B3(R392E) did not significantly change the percentage (Figure 7D). These results further support the mechanism that SH2B3 inhibits NGF signaling and neurite outgrowth through binding to TrkA, reduced TrkA phosphorylation, and reduced the interaction between SH2B1β and TrkA.

### SH2B3 inhibits axonal extension of cortical neurons

**Figure 8:**
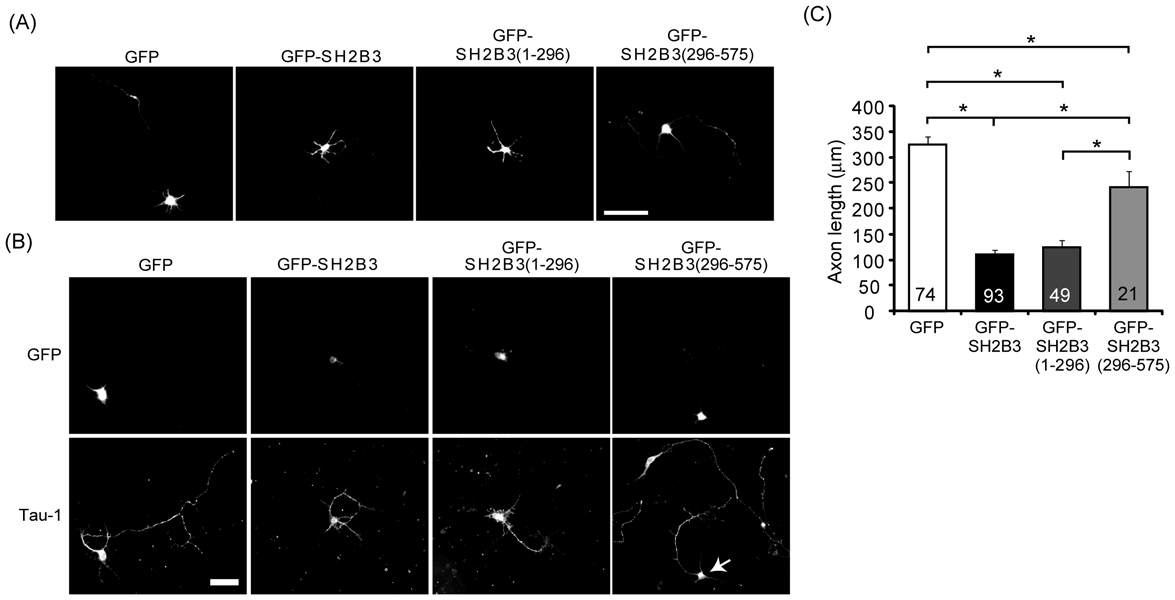
SH2B3 inhibits neurite extension of cortical neurons. (A) Isolated E18 rat cortical neurons were transfected with GFP, GFP-SH2B3, GFP-SH2B3(1–296), or GFP-SH2B3(296–575) on DIV 4. Images were taken on DIV 5. Scale bar: 50 µm. (B) E18 rat cortical neurons were treated as in (A). Transfected neurons were shown on the upper panels. Cells were subjected to immunofluorescence staining using anti-Tau-1 antibody and Alexa Fluor 555-conjugated secondary antibody (lower panels). Tau-1 is an axonal marker. Arrow points to GFP-SH2B3(296-575)-transfected cell. Images were taken using Zeiss Observer Z1 microscope. Scale bar: 50 µm. (C) E18 rat cortical neurons were treated as in (A). The averaged neurite length on DIV 5 was measured using Image J software. Total numbers of neurons analyzed are shown on the bars. *: P<0.05, one-way ANOVA.

We next determine whether SH2B3 also exerts inhibitory effect on the development of cortical neurons. Cortical neurons were isolated from E18 embryo, GFP, GFP-SH2B3, GFP-SH2B3(1–296), or GFP-SH2B3(296–575) was transiently transfected to DIV 4 neurons. Dendrites as well as axonal growth were monitored each day. Live cell images shown are from DIV 5. Neurons transfected with GFP showed normal dendrite morphology with one longest neurite being the axon. When GFP-SH2B3 was overexpressed, most neurites appeared as dendrite-like morphology and no axon was found (Figure 8A). As primary neurons are isolated and cultured *in vitro*, neurites are initiated spontaneously. So, one cannot test the effect of SH2B3 on neurite initiation. Similar to the results shown for PC12 cells, neurite elongation, specifically the axonal extension, was inhibited for cortical neurons that overexpressed GFP-SH2B3(1–296) (Figure 8B). Axonal marker, Tau-1, was used to identify axons. Neurons that overexpressed SH2B3(296–575) did not block axonal growth consistent with its role in affecting neurite initiation in PC12 cells (Figure 8A–B). The averaged axonal length of cortical neurons was around 325 µm for neurons expressing GFP, 120 µm for those expressing SH2B3 and SH2B3(1–296) and 250 µm for those that expressed SH2B3(296–575) (Figure 8C).

Taken together, this study presents the first characterization of the role of SH2B3 on neurite outgrowth of PC12 cells and cortical neurons. Unlike SH2B1β in enhancing neurotrophic factors-induced neurite outgrowth, overexpression of SH2B3 blocked it. Overexpressing SH2B3 significantly reduced NGF- and FGF1-induced signaling. We further demonstrated that the binding of SH2B3 to TrkA was required for the inhibitory effect. An interesting finding from this work suggests that the amino acids 1–296 of SH2B3 inhibit axonal elongation, whereas amino acids 296–575 inhibit neurite initiation.

## Discussion

In this study, we demonstrated that SH2B3 was expressed in the cerebral cortex of embryonic rat brain. In PC12 cells, the expression of SH2B3 was increased in response to NGF stimulation. However, overexpression of SH2B3 inhibited NGF-induced neurite outgrowth suggesting a novel function of SH2B3 in desensitizing NGF signaling. This result makes SH2B3 the only member of SH2B adaptor family capable of exerting a negative role on NGF signaling and neurite outgrowth. To understand the mechanism by which SH2B3 may exert its inhibitory effect, we showed that SH2B3 bound to activated TrkA. However, overexpressing SH2B3 reduced NGF-indcued pTrkA(Y490), reduced the interaction between SH2B1β and TrkA, and thus reduced NGF signaling and neurite outgrowth. Theses findings put SH2B3 as a feedback-induced antagonist of NGF- and FGF1-induced signaling, similar to Sprouty, Sef (similar expression to FGF) and Spred families [29], [30], [31], [32], [33], [34].

SH2B3 has previously been shown to negatively regulate signaling initiated by Epo receptor, interleukin-3 receptor, c-Kit, and Mpl [19], [21], [35], [36]. Given the various domains within SH2B3, it could potentially modulate downstream signaling in diverse ways. Results from the current study implicated that SH2B3 reduced the phosphorylation of TrkA at tyrosine 490 as well as the interaction between SH2B1 and TrkA. Phosphorylation of TrkA at tyrosine 490 recruits another adaptor Shc [8]. This result strongly implicates that overexpression of SH2B3 reduces the recruitment of Shc to TrkA-containing complexes. It is also possible that overexpression of SH2B3 may reduce the overall phosphorylation of TrkA. Phosphorylation of TrkA may contribute to the stability of dimerized TrkA allowing for the recruitment of other signaling proteins. Along this line, reduced binding of TrkA and PLCγ, as has been demonstrated for the inhibitory effect of SH2B2 on platelet-derived growth factor receptor B (PDGFRB) signaling [37]. SH2B3 could potentially regulate TrkA stability. Although both human and rat SH2B3 contain a binding site for c-Cbl, an ubiquitin ligase that functions to target proteins for degradation [38], human and rat SH2B1β do not (Figure S1). This difference may underlie the differential effect of hSH2B3 and rSH2B1β in inhibiting versus enhancing NGF signaling. For instance, in response to NGF, hSH2B3 may recruit c-Cbl to ubiquitinate TrkA, reduce its levels and thus desensitizes NGF signaling. Another possible mechanism is that SH2B3 negatively regulates TrkA-containing complexes by recruiting a putative phosphatase to de-phosphorylate pTrkA. This would then lead to reduced interaction between SH2B1β and TrkA without directly competing with SH2B1β for the same binding sites on TrkA.

SH2 domain is believed to interact with activated receptor tyrosine kinases. Nevertheless, a very recent report suggests that activation of PDGFRs is dispensable for the interaction with SH2B3 [39]. The authors hypothesized that the domain of SH2B3 flanked by DD and PH domains (contains three proline-rich regions) is responsible for phosphotyrosine-independent binding of SH2B3 to PDGFRs [39]. Our results from this work cannot exclude the possibility that SH2B3(1–296), containing DD, proline-rich and PH domains, could bind to TrkA with low affinity. This mechanism may explain our result that overexpressing SH2B3(1-296) did not block neurite initiation but blocked neurite elongation of PC12 cells and axonal extension of cortical neurons. Nonetheless, this result could also due to the inhibitory effect caused by the NGF-induced SH2B3 levels. Another possibility is that SH2B3(1–296) could dimerize with SH2B1 through DD domain, interfere with actin- and Rac-binding ability of SH2B1 and thus inhibit actin remodeling during neurite outgrowth. Amino acids 296-575 of SH2B3 on the other hand inhibit specifically neurite initiation of PC12 cells. As cortical neurons are isolated and cultured *in vitro*, neurites initiate spontaneously. Thus, the inhibitory effect of neurite initiation of cortical neurons by SH2B3(296–575) was not observed.

The main physiological outcome of suppressing growth factor or cytokine signaling by SH2B3 is reduced proliferation [17], [19], [21]. During NGF-induced neuronal differentiation in PC12 cells, increased proliferation at early phase is required [40]. Thus, inhibiting proliferation by SH2B3 could well be another mechanism of its inhibitory effect on neuronal differentiation. During embryonic development, tightly regulated balance between proliferation and differentiation controls cell fate. We think that different SH2B family members may serve to maintain this fine balance to assure normal development.

## Supporting Information

Figure S1 **Sequence alignment of the SH2 domains of rat SH2B1β and human SH2B3.** Sequences of the SH2 domains of rat SH2B1β (rSH2B1beta) and human SH2B3 (hSH2B3) were aligned using NCBI protein blast function (http://blast.ncbi.nlm.nih.gov/Blast.cgi). *: exact match between the two sequences. The recognition site for c-Cbl was marked.
(DOC)
