# Supplementary material for: The Adaptor Protein SH2B3 (Lnk) Negatively Regulates Neurite Outgrowth of PC12 Cells and Cortical Neurons
Source: PLoS One. 2011 Oct 18;6(10):e26433. doi: 10.1371/journal.pone.0026433 (PMC3196555; doi:10.1371/journal.pone.0026433)
Supplement: Figure S1 — Sequence alignment of the SH2 domains of rat SH2B1β and human SH2B3. Sequences of the SH2 domains of rat SH2B1β (rSH2B1beta) and human SH2B3 (hSH2B3) were aligned using NCBI protein blast function (http://blast.ncbi.nlm.nih.gov/Blast.cgi). *: exact match between the two sequences. The recognition site for c-Cbl was marked. (DOC) [file pone.0026433.s001.doc]

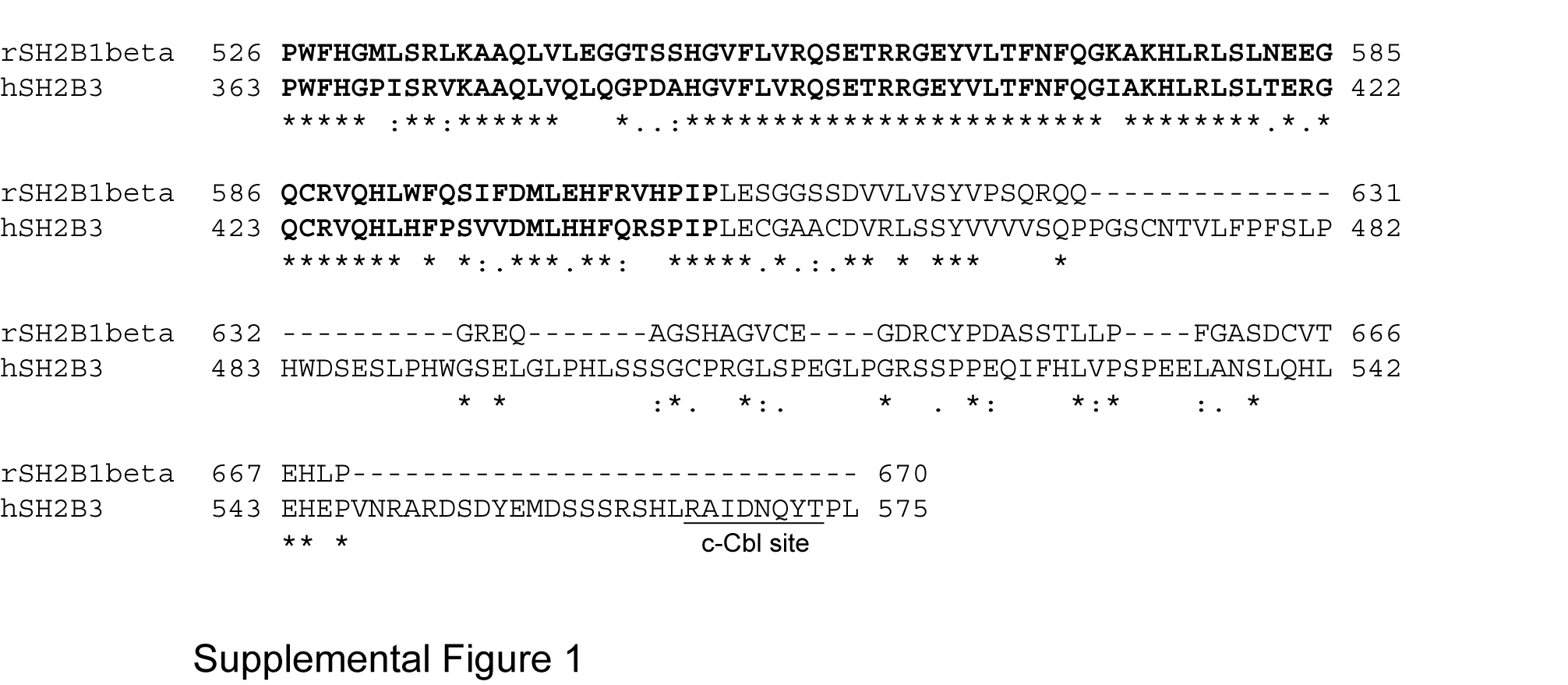


**Figure S1. Sequence alignment of the SH2 domains of rat SH2B1β and human SH2B3**. Sequences of the SH2 domains of rat SH2B1β (rSH2B1beta) and human SH2B3 (hSH2B3) were aligned using NCBI protein blast function (<http://blast.ncbi.nlm.nih.gov/Blast.cgi>). *: exact match between the two sequences. The recognition site for c-Cbl was marked.
